# Screening for FtsZ Dimerization Inhibitors Using Fluorescence Cross-Correlation Spectroscopy and Surface Resonance Plasmon Analysis

**DOI:** 10.1371/journal.pone.0130933

**Published:** 2015-07-08

**Authors:** Shintaro Mikuni, Kota Kodama, Akira Sasaki, Naoki Kohira, Hideki Maki, Masaharu Munetomo, Katsumi Maenaka, Masataka Kinjo

**Affiliations:** 1 Laboratory of Molecular Cell Dynamics, Faculty of Advanced Life Science, Hokkaido University, Sapporo, Japan; 2 Creative Research Institution, Hokkaido University, Sapporo, Japan; 3 Bio-Analytical Research Group, Biomedical Research Institute, National Institute of Advanced Industrial Science and Technology, Ibaraki, Japan; 4 Discovery Research Laboratory for Core Therapeutic Areas, Shionogi & Co., Ltd., Toyonaka, Osaka, Japan; 5 Information Initiative Center and Graduate School of Information Science and Technology, Hokkaido University, Sapporo, Japan; 6 Laboratory of Biomolecular Science, Faculty of Pharmaceutical Science, Hokkaido University, Sapporo, Japan; CNR, ITALY

## Abstract

FtsZ is an attractive target for antibiotic research because it is an essential bacterial cell division protein that polymerizes in a GTP-dependent manner. To find the seed chemical structure, we established a high-throughput, quantitative screening method combining fluorescence cross-correlation spectroscopy (FCCS) and surface plasmon resonance (SPR). As a new concept for the application of FCCS to polymerization-prone protein, *Staphylococcus aureus* FtsZ was fragmented into the N-terminal and C-terminal, which were fused with GFP and mCherry (red fluorescent protein), respectively. By this fragmentation, the GTP-dependent head-to-tail dimerization of each fluorescent labeled fragment of FtsZ could be observed, and the inhibitory processes of chemicals could be monitored by FCCS. In the first round of screening by FCCS, 28 candidates were quantitatively and statistically selected from 495 chemicals determined by *in silico* screening. Subsequently, in the second round of screening by FCCS, 71 candidates were also chosen from 888 chemicals selected via an *in silico* structural similarity search of the chemicals screened in the first round of screening. Moreover, the dissociation constants between the highest inhibitory chemicals and *Staphylococcus aureus* FtsZ were determined by SPR. Finally, by measuring the minimum inhibitory concentration, it was confirmed that the screened chemical had antibacterial activity against *Staphylococcus aureus*, including methicillin-resistant *Staphylococcus aureus* (MRSA).

## Introduction

**Fig 1:**
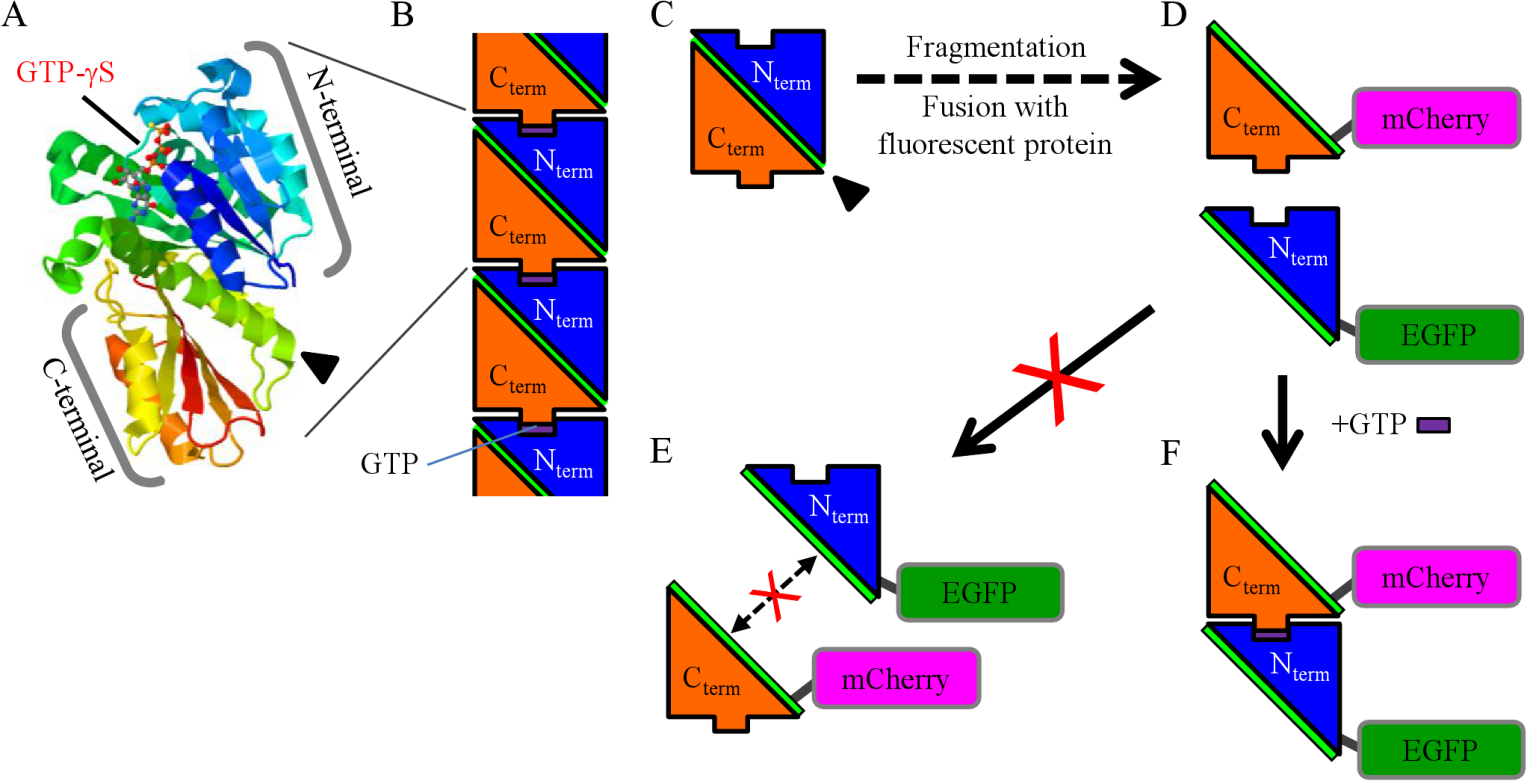
The structure and fragmentation of FtsZ. A. The crystal structure of the *S*. *aureus* FtsZ monomer bound to GTP-γS (Protein Data Bank: 3WGN). B. FtsZ polymerizes into tubulin-like protofilaments by head-to-tail association with GTP. C. FtsZ is shown schematically. The black arrowhead indicates the α-helix in the middle of FtsZ as shown in Fig 1A. D. For FCCS, FtsZ protein was fragmented. The N- and C-terminal FtsZ were fused with EGFP and mCherry (red fluorescent protein), respectively. The mutation at K175D in the N-terminal fragment was inserted to prevent further bundling FtsZ. E. To avoid interaction independent of GTP addition, the α-helix was retained in both the N- and C-terminal fragments (black arrowheads in A and C) in the middle of FtsZ structure. F. By binding to GTP, FtsZ^K175D^_N-terminal-EGFP and FtsZ_C-terminal-mCherry could be dimerized in a head-to-tail manner.

Cytokinesis in bacteria is achieved via protein assembly initiated by polymerization of the tubulin homologue filamenting temperature-sensitive mutant Z (FtsZ, Fig 1A) into the Z-ring, a ring-like structure that lies close to the cytoplasmic membrane at the prospective division site [1–3]. By binding to GTP, FtsZ polymerizes into tubulin-like protofilaments in head-to-tail association of individual units consisting of the C-terminal domain and N-terminal GTPase activation domain (Fig 1B) [4, 5]. Therefore, FtsZ could be a target for new antibiotics because it is the key protein of bacterial cell division. Chemical screening has been performed by filter-trapping [6] and monitoring the turbidity [7–11] and viability of bacteria [12–15] to evaluate the polymerization activity of FtsZ. One of the earliest-identified and well-investigated antibacterial agents against *S*. *aureus* is PC190723 [16], many derivatives of which have been synthesized to improve its antibacterial activity [17–20]. Moreover, the antibacterial mechanism of PC190723 functions via impairment of the recycling of FtsZ because the polymer of FtsZ is stabilized by PC190723 [21, 22]. However, it is important to develop new antibiotics from the viewpoint of destabilizing the polymer of FtsZ.

In this study, we developed a screening method combining fluorescence cross-correlation spectroscopy (FCCS) and surface plasmon resonance (SPR) to identify inhibitors of polymerization of FtsZ from a chemical library. FCCS is a prominent method to quantify not only biomolecular interactions from their cross-correlation functions, but also the diffusion time and number of fluorescent-labeled biomolecules from their autocorrelation functions [23–43]. By fragmentation of *S*. *aureus* FtsZ into N-terminal and C-terminal regions, and fusion of them with GFP and mCherry (red fluorescent protein), respectively (Fig 1C and 1D), the head-to-tail dimerization of each fluorescent labeled fragment of FtsZ could be observed by FCCS (Fig 1E and 1F). Therefore, the inhibitory processes of chemicals could be monitored by FCCS. To our knowledge, this is the first report on screening of chemicals targeting a polymerization-prone protein (in this case, FtsZ) by FCCS.

Moreover, to confirm specific interactions between *S*. *aureus* FtsZ and the chemical screened by FCCS, the dissociation constant (Kd) was determined by SPR. Finally, we found the chemical that were demonstrated to have antibacterial activity.

## Materials and Methods

### Chemicals and bacterial strains

For FCCS screening, all chemicals were from the Open Innovation Center for Drag Discovery of The University of Tokyo. They were used in DMSO solutions. For SPR measurement, chemicals were purchased from Enamine Ltd. (Kiev, Ukraine), Vitas-M Laboratory (Narva, Estonia) and Pharmeks, Ltd. (Moscow, Russia), and used as DMSO solutions. GTP was purchased from WAKO (Japan) and used as a solution in 50 mM Tris-HCl buffer (pH 8.0). Doripenem was provided by Shionogi & Co., Ltd.

Bacterial strains were purchased from the American Type Culture Collection (ATCC). The other tested strains were described previously [44, 45].

### High-throughput virtual screening

A docking algorithm, AutoDock 4.0, was used for *in silico* screening. The chemical library of the Open Innovation Center for Drug Discovery (The University of Tokyo) consisting of approximately 210,000 individual structures was prepared using the LigPrep task to produce structural variations, perform corrections, exclude undesirable structures, generate tautomers, add hydrogen atoms, neutralize charged groups and optimize ligand structures for a pH range from 5 to 9.

For the construction of a grid for screening by the use of the DOCK4.0 program, the grid box was set directly on the two cavities of the FtsZ crystal structure 4DXD from the PDB database (www.pdb.org) after extraction of PC190723, which is known to have efficiency against the FtsZ and GDP molecules. Molegro Virtual Docker was used for cavity detection. One of the selected cavities was the PC190723 binding site.

The extracted cavities were covered with the grid box. For the high-throughput virtual screening, parallel virtual screening was used so that modeFRONTIER (ESTECO) submitted the job to AutoDock in a Linux-based computer cluster. Each prepared 3D ligand structure was docked to the two cavities, and the docking score was calculated. The virtual screening was carried out on an XL server (total of 40 cores and 256GB memory) of the Hokkaido university academic cloud provided by the Information Initiative Center of Hokkaido University (http://www.iic.hokudai.ac.jp/).

### Purification of FtsZ protein

The sequences coding N- and C-terminal *S*. *aureus* FtsZ were amplified by PCR, fused with EGFP and mCherry (red fluorescent protein), respectively, and inserted into pRSET vector (Invitrogen). A mutation was constructed at K175D to prevent further bundling of FtsZ in the N-terminal fragment. Moreover, the α-helix regions of the N- and C-terminal fragments were preserved (indicated in light green) in the middle of the FtsZ structure as shown in Fig 1A and 1C. The proteins of FtsZ^K175D^_N-terminal-EGFP and FtsZ_C-terminal-mCherry were expressed in *E*. *coli*, BL21 pLysS (Invitrogen), and incubated at 15°C for 20 hours with 0.5 mM IPTG. After harvest, cells were lysed by sonication in a buffer consisting of 50 mM Tris-HCl (pH 8.0), 250 mM KCl and 20 mM imidazol supplemented with the following protease inhibitors: 50 μM bestatin (ACROS, Inc.), 800 μM AEBSF (Santa Cruz), 15 μM E-64, 20 μM pepstatinA, 7 μM phosphoramidon, and 10 μM leupeptin (Peptide Institute, INC) on ice. The cell lysate was recovered by centrifugation at 4°C. After filtration, the lysate was purified using AKTAprime plus (GE Healthcare) installed in HisTrap HP and HiTrap Q HP columns (GE Healthcare) for Ni affinity and anion-exchange chromatography, respectively. For Ni affinity chromatography, equilibrium buffer (50 mM Tris-HCl [pH 8.0], 250 mM KCl, 20 mM imidazol and 10% glycerol), and elution buffer (50 mM Tris-HCl [pH 8.0], 250 mM KCl, 500 mM imidazol and 10% glycerol) were used. After Ni affinity chromatography, the fractions containing his-tagged proteins were collected and desalted using Amicon Ultra 30K filters (Merck Millipore). After filtration of the desalted solution, anion-exchange chromatography was performed using equilibrium buffer (50 mM Tris-HCl [pH 8.0], 50 mM KCl and 10% glycerol) and elution buffer (50 mM Tris-HCl [pH 8.0], 1 M KCl and 10% glycerol). The fractions containing FtsZ^K175D^_N-terminal-EGFP and FtsZ_C-terminal-mCherry were collected and desalted using an Amicon Ultra 30K filters (Merck Millipore). The concentrations of purified proteins were determined by FCS.

### Measurement of fluorescence cross correlation spectroscopy (FCCS)

The conditions and system used for FCCS measurements were described in a previous report [41]. FCCS measurement was carried out for 15 s in this experiment.

The purified FtsZ^K175D^_N-terminal-EGFP and FtsZ_C-terminal-mCherry were mixed in 0.44–0.85 μM each in a buffer composed of 50 mM Tris-HCl [pH 8.0], 500 mM Na_2_SO_4_ and 5 mM MgCl_2_. Mixed samples were dispensed as 48.5 μL aliquots into a 384-well glass bottom microplate (Olympus) and a 0.5 μL aliquot of the chemical at a concentration of 2 or 10 mM was added to each well. The final concentration of the chemicals was 20 or 100 μM. After mixing, FCCS measurement was performed (premeasurement, data not shown). After premeasurement of FCCS, 1 μL of GTP (100 mM) was added to each well (final concentration: 2 mM). After mixing and 30 min incubation at room temperature, FCCS measurement was performed. Using samples purified on a different day, independent screening was performed 3 times.

### Data analysis of FCCS measurements

The data acquired from FCCS were analyzed using ZEN software (Zeiss, Germany). The fluorescence autocorrelation functions from the green, *G*_*G*_ *(τ)*, and red channels, *G*_*R*_ *(τ)*, and the fluorescence cross-correlation functions, *G*_*C*_ *(τ)* were calculated according to the normalized correlation function.

$$G_{G}(\tau )=1+\frac{\left\langle \delta I_{G}(t)\cdot \delta I_{G}(t+\tau )\right\rangle }{\left\langle I_{G}(t)\rangle \cdot \langle I_{G}(t)\right\rangle }$$ (1)

$$G_{R}(\tau )=1+\frac{\left\langle \delta I_{R}(t)\cdot \delta I_{R}(t+\tau )\right\rangle }{\left\langle I_{R}(t)\rangle \cdot \langle I_{R}(t)\right\rangle }$$ (2)

$$G_{C}(\tau )=1+\frac{\left\langle \delta I_{G}(t)\cdot \delta I_{R}(t+\tau )\right\rangle }{\left\langle I_{G}(t)\rangle \cdot \langle I_{R}(t)\right\rangle }$$ (3)

where *τ* denotes the time delay, *I*_*G*_ is the fluorescent intensity of the green channel, and *I*_*R*_ is the fluorescent intensity of the red channel. The acquired autocorrelations were fitted using a two-component model, and cross-correlation was fitted using a one-component model.

$$G(\tau )=1+\frac{1-F_{triplet}+F_{triplet}\exp\left(-\tau /\tau_{trplet}\right)}{N(1-F_{triplet})}\times \left(\left(\frac{F_{fast}}{1+\tau /\tau_{fast}}\right)\sqrt{\frac{1}{1+\tau /s^{2}\tau_{fast}}}+\left(\frac{F_{slow}}{1+\tau /\tau_{slow}}\right)\sqrt{\frac{1}{1+\tau /s^{2}\tau_{slow}}}\right)$$ (4)

where *F*_*triplet*_ is the average fraction of triplet state molecules (in the fitting of cross-correlation, assuming *F*_*triplet*_ is 0), *τ*_*triplet*_ is the triplet relaxation time, and *F*_*fast*_ and *F*_*slow*_ are the fractions of the fast and slow components, respectively. *τ*_*fast*_ and *τ*_*slow*_ are the diffusion times of the fast and slow components, respectively. In the fitting of *G*_*G*_ *(τ)*, *τ*_*fast*_ was fixed in the range from 100 to 200 μs. In the fitting of *G*_*R*_ *(τ)*, *τ*_*fast*_ was fixed in the range from 200 to 300 μs. *N* is the average number of fluorescent particles in the excitation-detection volume defined by radius *w*, *z* is half of the long axis of the confocal volume element, and *s* is the structural parameter representing the ratio *s* = *z*/*w*. The values of *w*_*i*_ (*i = G or R*) are determined from the diffusion coefficients of the rhodamine 6G and Alexa Fluor 594 used as standard dyes, respectively.

The average number calculated from autocorrelation *N*_*G*_ and *N*_*R*_ and complex cross-correlated particles *N*_*C*_ is given by

$$N_{G}=\frac{1}{G_{G}(0)-1}$$ (5)

$$N_{R}=\frac{1}{G_{R}(0)-1}$$ (6)

$$N_{C}=\frac{G_{C}(0)-1}{\left(G_{R}(0)-1)\cdot (G_{G}(0)-1\right)}$$ (7)

The binding ratio (*BR*), which directly indicates the tendency of interaction between FtsZ^K175D^_N-terminal-EGFP and FtsZ_C-terminal-mCherry, was defined by the following Eq (8). When a chemical has a higher inhibitory effect, *BR* should be lower.

$$BR=\frac{N_{C}}{N_{G}}$$ (8)

Moreover, *inhibition %* was calculated using the following Eq (9). The *inhibition %* should be higher when the chemical has a higher inhibitory effect.

$$inhibition\;\%=\frac{BR_{DMSO,GTP(+)}-BR_{chemical,GTP(+)}}{BR_{DMSO,GTP(+)}}\times 100$$ (9)

where *BR*_DMSO,GTP(+)_ is the binding ratio in the presence of DMSO (vehicle) and GTP is the control value indicating no inhibition, *BR*_*chemical*,*GTP(+)*_ is the binding ratio in the presence a chemical and GTP. Considering the background, Eq (9) can be shown as follows.

$$\begin{array}{l} inhibition\;\%=\frac{\left(BR_{DMSO,GTP(+)}-BR_{background})-(BR_{chemical,GTP(+)}-BR_{background}\right)}{BR_{DMSO,GTP(+)}-BR_{background}}\times 100\\ \;=\frac{BR_{DMSO,GTP\left(+\right)}-BR_{chemical,GTP\left(+\right)}}{BR_{DMSO,GTP\left(+\right)}-BR_{background}}\times 100 \end{array}$$ (10)

where *BR*_*background*_ is the binding ratio of the background obtained in the absence of GTP.

To evaluate the quality of screening, the *Z’-factor* was estimated as follows [46]

$$Z\prime \text{-}factor=1-\frac{3\sigma_{DMSO,GTP(+)}+3\sigma_{DMSO,GTP(-)}}{\mu_{DMSO,GTP(+)}-\mu_{DMSO,GTP(-)}}$$ (11)

where *σ*_DMSO,GTP(+)_ and *σ*_DMSO,GTP(-)_ are standard deviations of the binding ratio obtained in the presence and absence of GTP, respectively, and with DMSO as the vehicle, *μ*_DMSO,GTP(+)_ and *μ*_DMSO,GTP(-)_ are the averages of the binding ratio obtained in the presence and absence of GTP, respectively, with DMSO as the vehicle. The average and standard deviation were calculated using more than 9 data sets.

### Measurement and analysis of surface plasmon resonance (SPR)

SPR measurements were performed using a Biacore T200 (GE Healthcare). The full length *S*. *aureus* FtsZ fused with mCherry (FtsZ-FL-mCherry) was diluted to 5 μM with acetate buffer (pH 4.0) and fixed on a CM5 sensor chip (GE Healthcare) using an Amine Coupling Kit (GE Healthcare). FtsZ-FL-mCherry was purified by Ni affinity and anion-exchange chromatography using the same method as for fragmented FtsZs (Materials and methods *Purification of FtsZ protein*), by size-exclusion chromatography using a Superdex 200 10/300 GL column (GE Healthcare) installed in an AKTAprime plus (GE Healthcare). For size-exclusion chromatography, PBS with 0.05% Tween-20 was used as the running buffer. For detecting the interactions between chemicals and FtsZ-FL-mCherry, PBS with 0.05% Tween-20 and 1% DMSO was used as the basal buffer. The values of the binding response (*R*_*eq*_) obtained from SPR measurements of various concentrations of each chemical were analyzed and exported using T200 evaluation software (GE Healthcare). For determination of *Kd*, the value of the *theoretical R*_*max*_ of each chemical was calculated by Eq (12).

$$theoretical\;R_{\max}=MW_{chemical}\times \frac{R_{FtsZ-FL-mCherry}}{MW_{FtsZ-FL-mCherry}}$$ (12)

where *MW*_*chemical*_ and *MW*_*FtsZ-FL-mCherry*_ are the molecular weight of each chemical and FtsZ-FL-mCh, respectively, and *R*_*FtsZ-FL-mCherry*_ is the binding response corresponding to the amount of FtsZ-FL-mCh on the CM5 sensor chip. The *Kd* was calculated by fitting on the scatter plot of *R*_*eq*_ */theoretical R*_*max*_ versus the corresponding concentration of each chemical by Eq (13) using Origin software (OriginLab Corporation).

$$R_{eq}/theoretical\;R_{\max}=\frac{c}{Kd+c}\times a+b$$ (13)

where *R*_*eq*_ is the binding response obtained at the concentration (*c*) of each chemical, *a* is *R*_*max*_ */theoretical R*_*max*_, and *b* is *offset*/*theoretical R*_*max*_. In this fitting analysis, the upper limit of *a* was set as 1.

### Antibiotic susceptibility test

Antimicrobial susceptibility testing was performed by broth microdilution in cation-adjusted Mueller–Hinton broth (CAMHB) according to the CLSI guidelines [47]. Overnight culture was suspended in CAMHB, and the OD_625_ was measured using an Ultrospec 6300 pro spectrophotometer (GE Healthcare). The culture suspension was diluted to 0.01 of the OD_625_, and 5 μL of it was inoculated into test medium containing the test chemical. Then incubation was performed at 35°C for 16 to 20 hours. The final inoculum size corresponded to ca. 5 x 10^4^ CFU per well, which corresponded to ca. 5 x 10^5^ CFU/mL. The minimum inhibitory concentration (MIC) endpoint was defined as the lowest concentration of the test chemical that inhibited bacterial growth as detected by the unaided eye.

## Results and Discussion

### High-throughput virtual screening

The X-ray crystal structure of the *S*. *aureus* FtsZ domain in the active configuration (PDB accession code 4DXD) was used as the target structure in this approach. On the basis of the results for the AutoDock scoring functions, the molecules were ranked, and the top 500 molecules were extracted and carefully considered for their receptor binding and scaffold diversity. We finally obtained 495 available candidate chemicals from the chemical library of the Open Innovation Center for Drug Discovery (The University of Tokyo) for the *in vitro* assay.

### FCCS screening

For FCCS measurement, *S*. *aureus* FtsZ was divided into N- and C-terminal fragments that were then fused with EGFP and mCherry (red fluorescent protein), respectively (Fig 1D). To prevent undesirable bundling of FtsZ, the lysine at 175 aa in the N-terminal fragment was replaced with aspartic acid [48]. Moreover, to avoid native reformation as shown in Fig 1E, the α-helical region (black arrowhead in Fig 1A and 1C) was preserved in both the C-terminal and N-terminal fragments as an insulator. Thus the FtsZ^K175D^_N-terminal-EGFP and FtsZ_C-terminal-mCherry could only be dimerized in head-to-tail form by adding GTP (Fig 1F).

**Fig 2:**
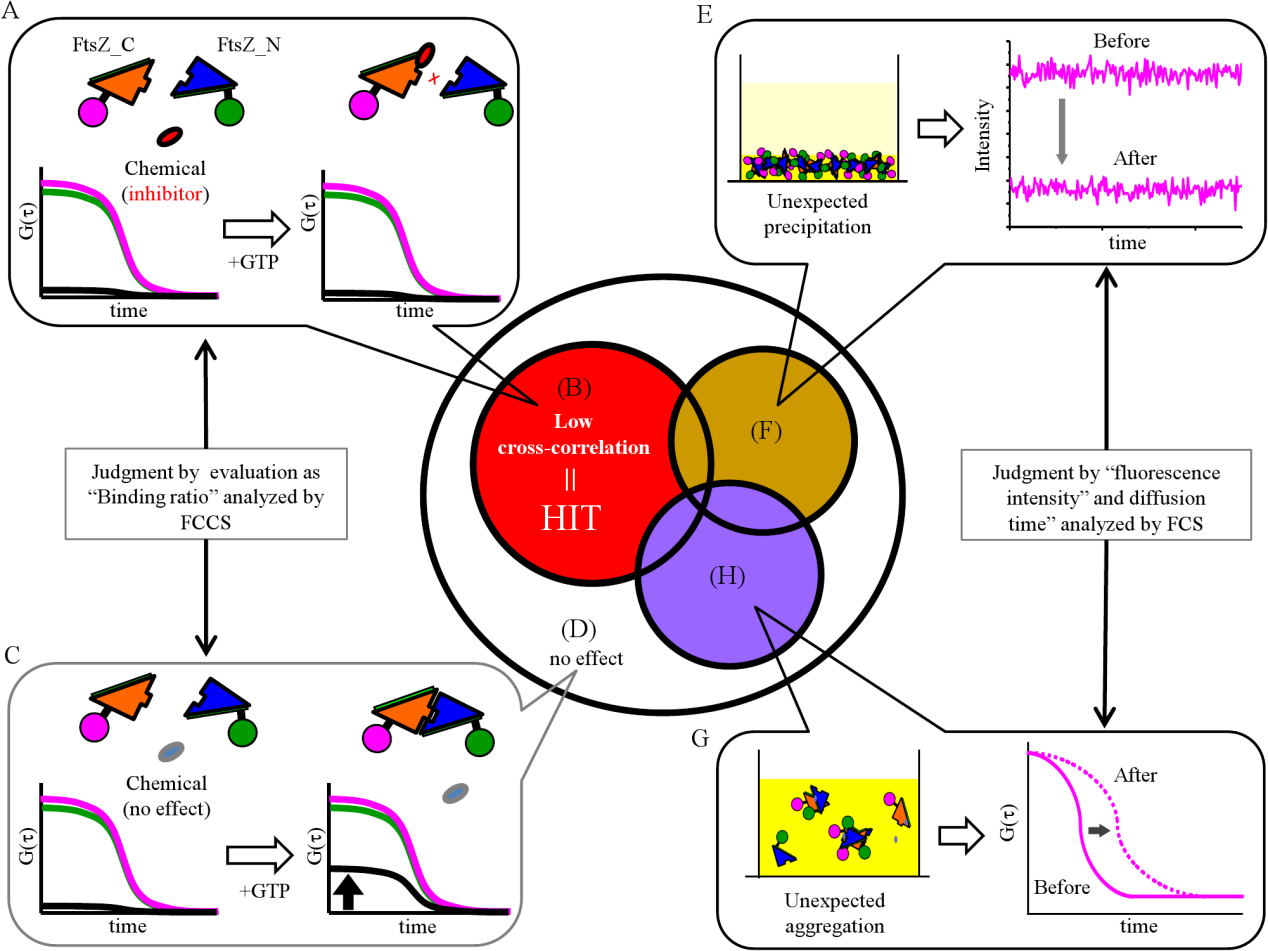
Strategic judgment of “Hit” chemicals by FCCS. A. When a chemical has an inhibitory effect on FtsZ dimerization even in the presence of GTP, the cross-correlation amplitude remains low. These inhibitory chemicals are included in the subset in the red circle (B). C. After addition of GTP, cross-correlation amplitude is increased if the chemical does not affect FtsZ (D). A and C were evaluated by the “binding ratio” analyzed by FCCS. The magenta and green lines indicate the autocorrelation functions of FtsZ^K175D^_N-terminal-EGFP and FtsZ_C-terminal-mCherry, respectively. The black line indicates the cross-correlation function. E. Fluorescent intensity (EGFP and/or mCherry) changed when FtsZ fragments were precipitated by addition of a chemical. Chemicals that induced intensity changes of more than 20% when DMSO was added are included in the subset in the brown circle (F). G. Diffusion of FtsZ fragments was slower by aggregation. Chemicals that increased the slow fraction by more than 20% in 2-component fitting are included in the subset shown in the purple circle (H). Finally, the chemicals included in the subset shown only in red, were determined to be “true Hit” chemicals.

In chemical screening, inhibition by chemicals was judged via reduction of cross-correlation amplitude (*Gc*(0)). As shown in Fig 2, if a chemical has an inhibitory effect on FtsZ dimerization, the cross-correlation amplitude should remain low even after addition of GTP (Fig 2A and 2B). In contrast, if the chemical has no effect on FtsZ dimerization, the cross-correlation amplitude should increase after addition of GTP (Fig 2C and 2D). The inhibitory effect was statistically evaluated as the “BR (binding ratio)” from 3 independent FCCS screenings. Chemicals with inhibitory effects on the binding ratio compared with the control (DMSO solution) were categorized into “Hit” as shown in the red circle in the Venn diagram in Fig 2. Moreover, to improve the precision of screening, inappropriate data such as unexpected aggregation and/or precipitation of the fragmented FtsZ were excluded from the subset “Hit” (Fig 2E and 2G). The exclusion criteria were a reduction in intensity of more than 20% from the average intensity of the control (DMSO solution) as precipitation (Fig 2E and 2F), and an increase of more than 20% of the slow fraction by 2-component fitting in diffusion analysis as aggregation (Fig 2G and 2H). Finally, the chemicals included in the subset of the area shown in red were determined to be “true Hit” chemicals.

**Fig 3:**
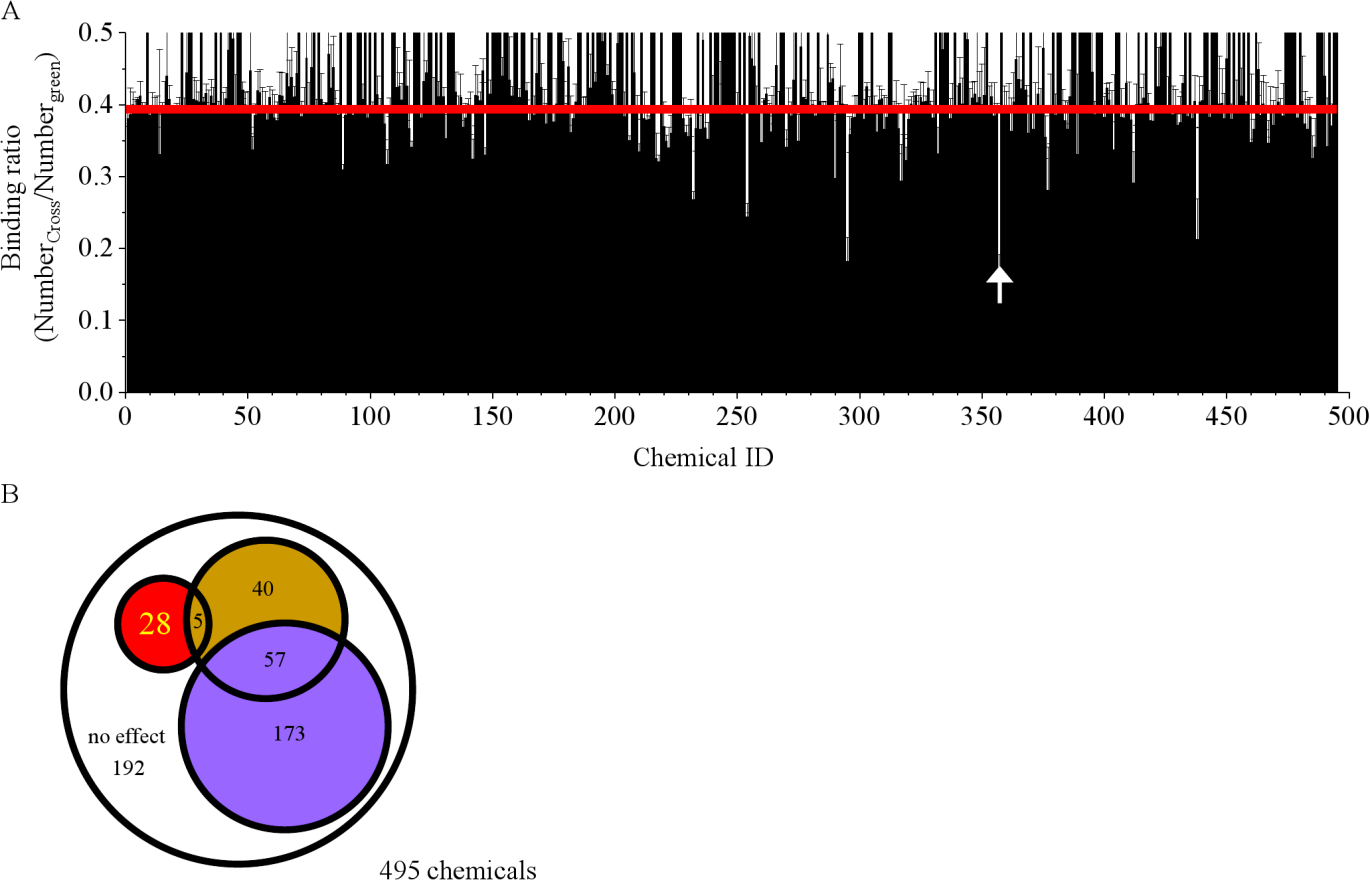
Results of 1st FCCS screening of 495 chemicals. A. Bar graph of averaged binding ratios from 3 independent screenings. Error bar indicates the standard deviation (n = 3). The red line indicates the average binding ratio obtained after addition of DMSO and GTP (= 0.390). The white arrow indicates the chemical that most effectively inhibited FtsZ dimerization. B. The Venn diagram shows a summary of the 1st screening by FCCS.

In the first round of FCCS screening, 495 candidate chemicals were selected by *in silico* screening and the obtained binding ratios and the results of the judgement are shown in Fig 3A and 3B, respectively. The Z’-factor of the first round of screening was 0.541. Twenty-eight chemicals were determined to be “true Hit” chemicals.

**Fig 4:**
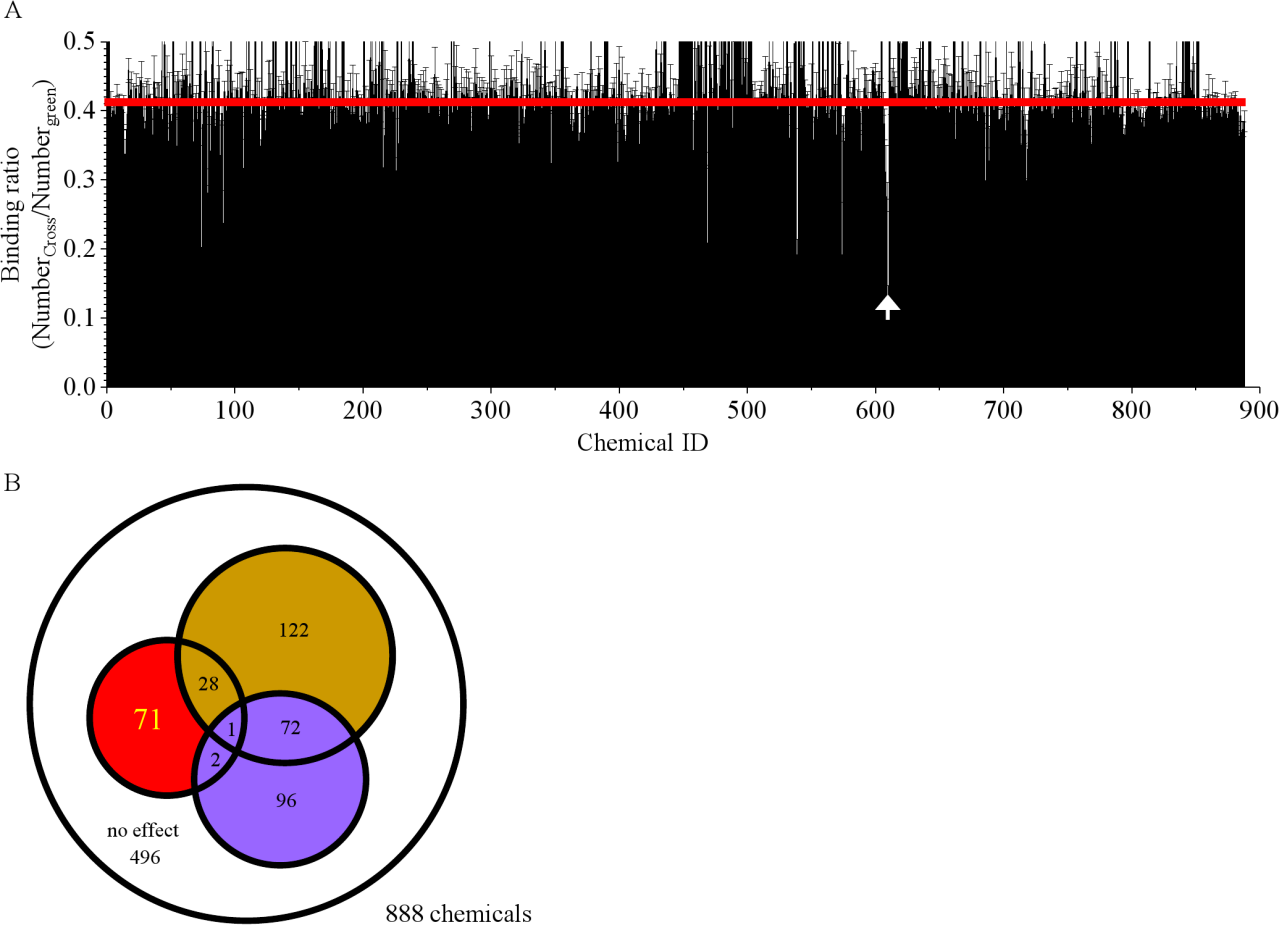
Results of 2nd FCCS screening of 888 chemicals. A. Bar graph of averaged binding ratios of 3 independent screenings. Error bar indicates the standard deviation (n = 3). The red line indicates the average binding ratio obtained after addition of DMSO and GTP (= 0.411). The white arrow indicates the chemical that most effectively inhibited FtsZ dimerization. B. The Venn diagram shows a summary of the 2nd screening results.

**Fig 5:**
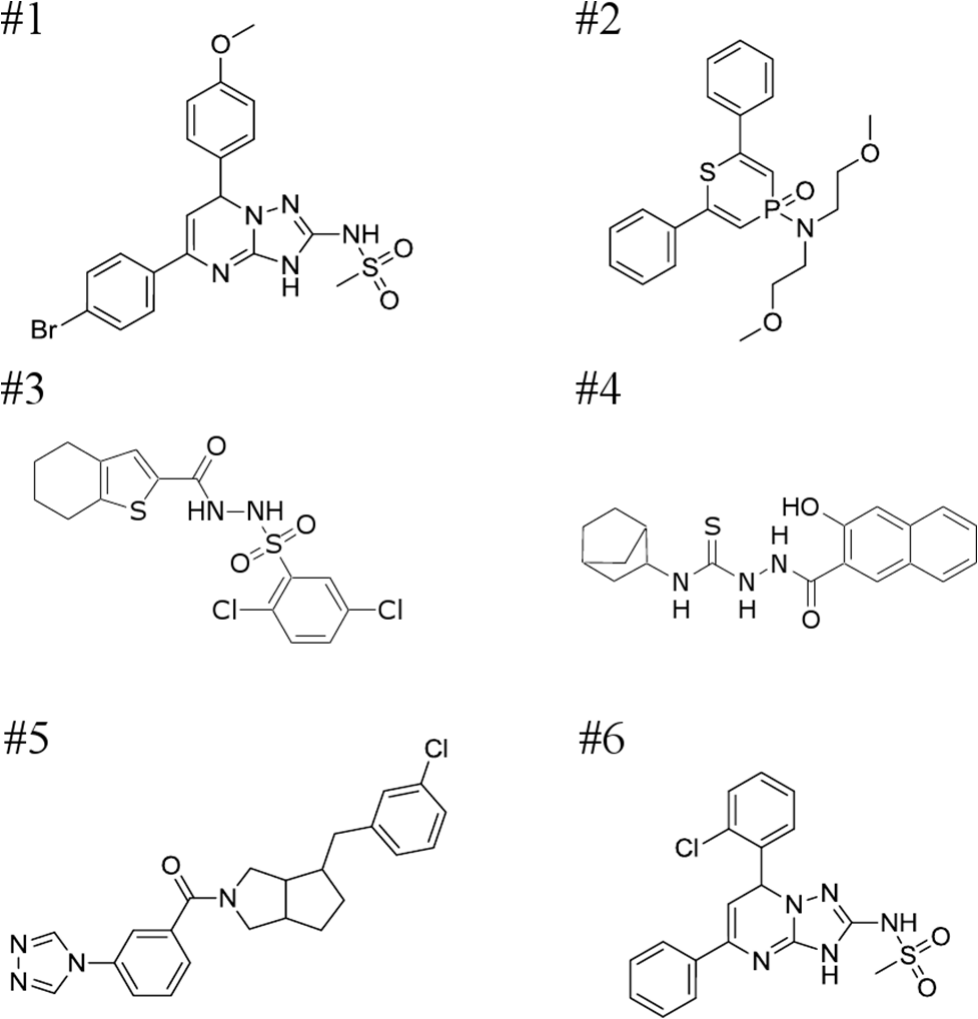
The structures of inhibitory chemicals. The chemicals are numbered from #1 to #6 in order of the inhibition % calculated from FCCS analysis.

On the basis of structural similarities to the 28 candidate chemicals chosen in the first round of FCCS screening, 888 chemicals were selected as further inhibitor candidates. The binding ratios obtained and the results of judgement from the subsequent FCCS screening of these 888 chemicals are shown in Fig 4A and 4B. The Z’-factor of the second screening was 0.623. Finally, 71 chemicals were determined to be “true Hits” and the 6 chemicals with the highest inhibitory effects are shown in Fig 5. The white arrow in Fig 4A indicates the lowest binding ratio obtained by addition of chemical #610, in other words, the one that had highest inhibitory effect on dimerization of FtsZ in the second screening. The measured auto- and cross-correlation functions for #610 are shown in S1 Fig. An enlargement of the area of the graph of Fig 4 around #610 is shown in S2A Fig. Chemicals #607, #608, and #609 also had lower binding ratios than the control (red line in S2A Fig). Interestingly, the core structures of #607, #608, #609 and #610 were similar, as shown in S2B Fig. This suggested that the core structure of these chemicals was important for binding to FtsZ.

### Dissociation constant between FtsZ and selected chemicals

**Fig 6:**
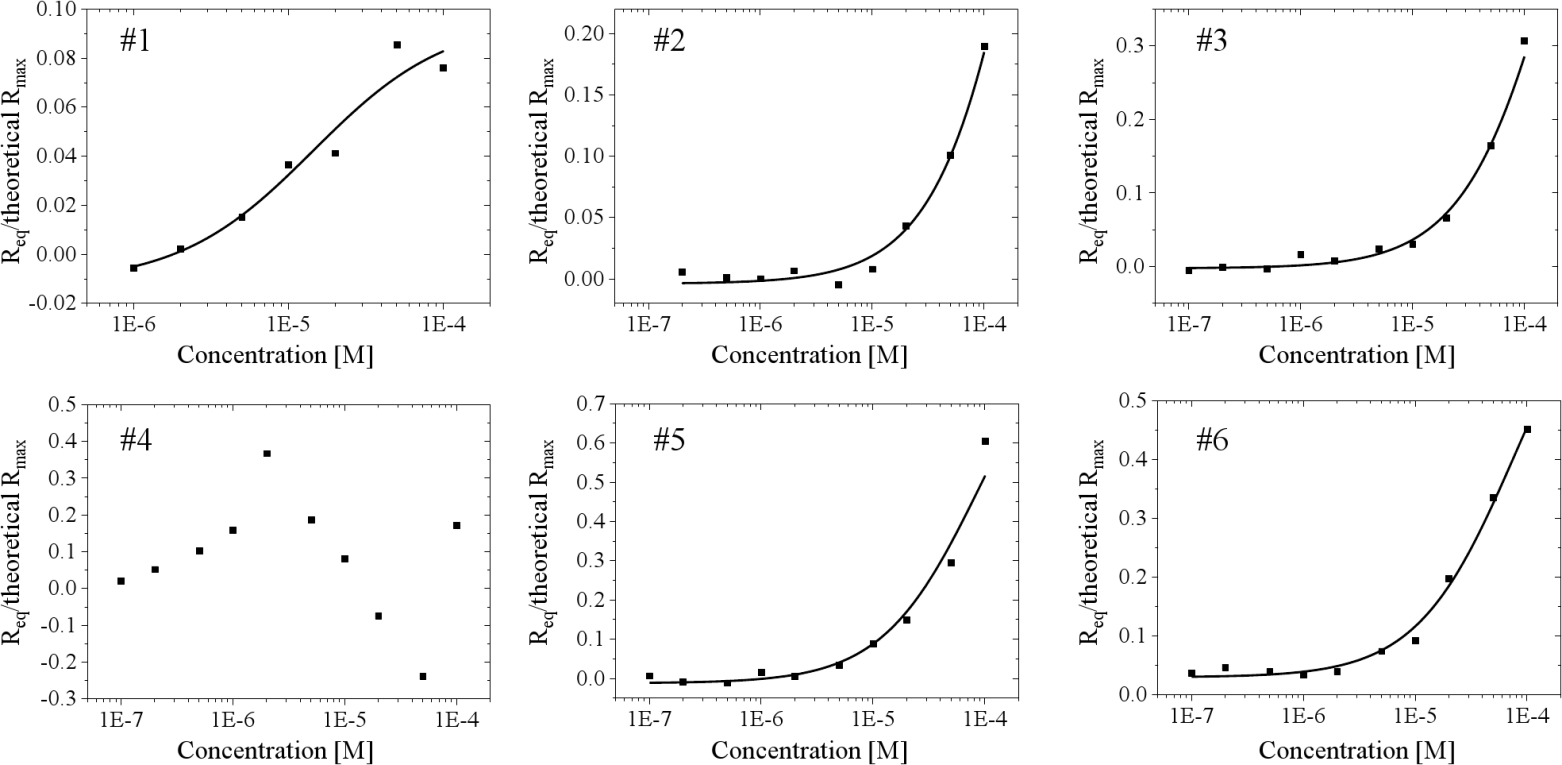
Typical scatter plot of R_eq_/theoretical R_max_ versus concentrations of chemicals and fitting. The filled squares represent the values of R_**eq**_/theoretical R_**max**_ at corresponding concentrations of each chemical. The black lines represent fitting on the points. Fitting analysis was performed using Origin software.

**Fig 7:**
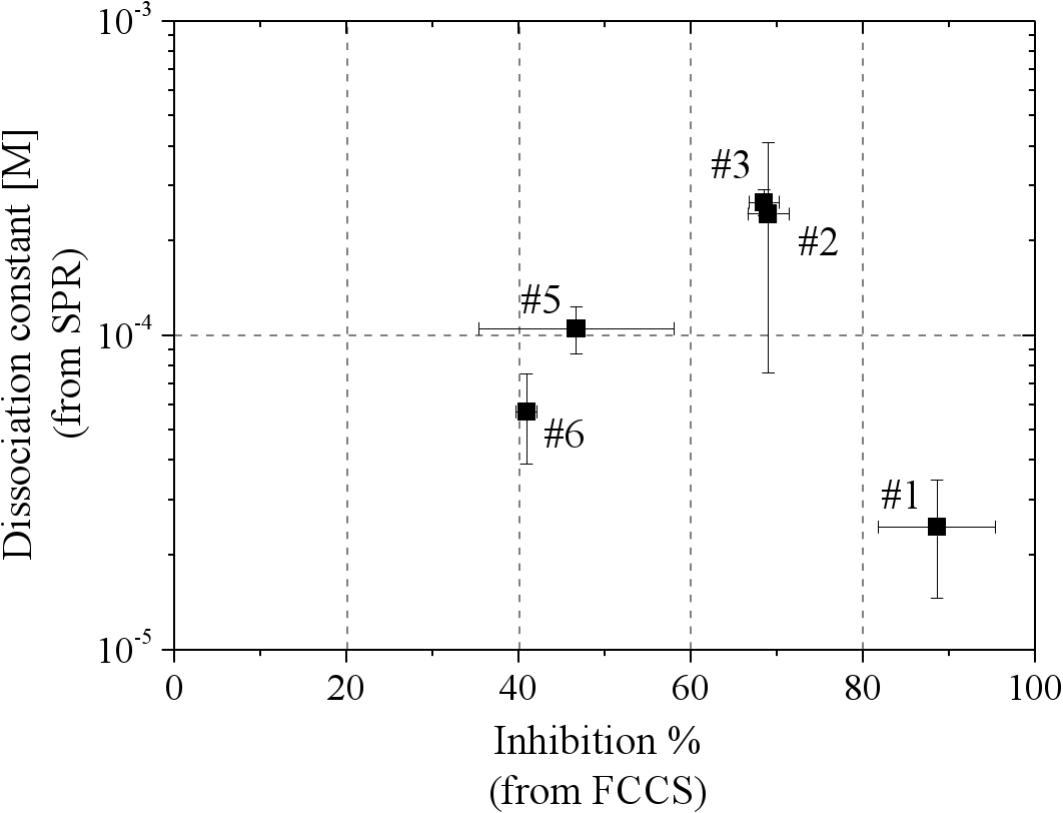
Relationship between inhibition % and dissociation constant (Kd).

To confirm whether the 6 chemicals shown in Fig 5 bound to FtsZ specifically, the dissociation constant (Kd) was determined by surface plasmon resonance (SPR). Fig 6 shows a scatter plot of R_eq_/theoretical R_max_ as a function of the concentrations of the chemicals. The values of R_eq_ were obtained from SPR measurement at various concentrations of each chemical. For the chemical #4, fitting analysis could not be properly performed because the values of R_eq_ decreased depending on the concentration of chemical #4 in the range higher than 5 μM. This decrease might have been caused by low solubility of chemical #4 in the aqueous buffer. The low solubility might have evoked nonspecific interaction between FtsZ and chemical #4. The relationship between the inhibition % (from FCCS) and Kd (from SPR) is summarized in Fig 7, Table 1 and S3 Fig. The most inhibitory chemical, #1, selected from FCCS screening had the lowest Kd value (Fig 7 and Table 1).

**Table 1:** Inhibition % and dissociation constant (Kd).

	Chemical					
	#1	#2	#3	#4	#5	#6
inhibition %	88.6 (6.8)	69.0 (2.4)	68.5 (1.7)	64.4 (5.8)	46.7 (11.4)	40.9 (1.2)
Kd [M]	2.45 (1.00) e -5	2.43 (1.67) e-4	2.65 (2.25)3-1	nd	1.05 (0.14) e-4	5.71 (1.82) e-5

nd: no data mean (SD), n = 3

### Antibiotic susceptibility test

The antibacterial activities of the chemicals that recognized the specific binding determined by SPR were examined by measuring the minimum inhibitory concentrations (MICs) of these chemicals against a panel of type strains of Gram-positive and Gram-negative bacterial pathogens. The results are summarized in Table 2. Chemical #2 inhibited the growth of *Staphylococcus* spp. and *Enterococcus* spp. Chemical #2 had no activity against other Gram-positive species or any of the Gram-negative pathogens tested. Other tested chemicals had no activity against any species. The MIC of chemical #2 against this panel of *S*. *aureus* ATCC 29213 (methicillin-susceptible *S*. *aureus*, MSSA) and *S*. *aureus* SR3637 (methicillin-resistant *S*. *aureus*, MRSA) was 8 μg/mL. The MIC of doripenem is 0.063 μg/mL or less for *S*. *aureus* ATCC 29213 and 16 μg/mL for *S*. *aureus* SR3637. For *E*. *faecalis*, the MIC of chemical #2 was 32 μg/mL. Thus, chemical #2 had antimicrobial activity against *E*. *faecalis* and *S*. *aureus*, including MRSA.

**Table 2:** Minimal inhibitory concentration (μg/mL) by antibiotic sensitivity test.

	Chemical						Doripenem
	#1	#2	#3	#4	#5	#6	
*S*. *aureus* ATCC 29213(MSSA*)	>64	8	>64	>32	>64	>64	≦0.063
*S*. *aureus* SR3637(MRSA**)	>64	8	>64	>32	>64	>64	16
*E*. *faecalis* ATCC 29212	>64	32	>64	>32	>64	>64	1
*E*. *faecalis* SR7914 (VRE***)	>64	32	>64	>32	>64	>64	8
*E*. *faecium* ATCC 19434	>64	>64	>64	>32	>64	>64	8
*E*. *faecium* SR7917 (VRE***)	>64	>64	>64	>32	>64	>64	64
*S*. *pneumoniae* Type I	>64	64	>64	>32	>64	>64	≦0.063
*E*. *coli* ATCC 25922	>64	>64	>64	>32	>64	>64	≦0.063
*K*. *pneumoniae* ATCC 13883	>64	>64	>64	>32	>64	>64	0.125
*P*. *aeruginosa* ATCC 27853	>64	>64	>64	>32	>64	>64	0.5
*A*. *baumannii* ATCC 19606	>64	>64	>64	>32	>64	>64	1
*H*. *influenzae* ATCC 10211	>64	>64	>64	>32	>64	>64	0.125

*MSSA: methicillin-susceptible *Staphylococcus aureus*.

**MRSA: methicillin-resistant *Staphylococcus aureus*.

***VRE: vancomycin-resistant *Enterococcus*.

## Conclusion

FtsZ is an essential protein for cell division of bacteria. Thus, inhibitors of dimerization, over-oligomerization and polymerization of FtsZ could be novel antibiotics. To find inhibitor(s) of dimerization of *S*. *aureus* FtsZ, we established a high-throughput, quantitative screening assay method combining fluorescence cross-correlation spectroscopy (FCCS) to search for inhibitors of dimerization and surface plasmon resonance (SPR) for evaluation of the specific binding of chemicals to the protein. The combination FCCS and SPR enabled us to conduct an effective chemical search since the methods have complementary aspects for searching and evaluation properties in screening.

Based on the protein-protein interaction model, we developed a new screening model for polymerization of a protein using fragments of the protein to prevent further polymerization or bundling. Thus, this screening method can be applied to proteins such as tubulin and actin, and can be expanded to aggregation-prone proteins like amyloid beta, huntingtin and α-synuclein for degenerative neurological disorder diseases [49].

## Supporting Information

S1 Fig The auto- and cross-correlation functions. The green and magenta lines represent the autocorrelation functions of FtsZ^K175D^_N-terminal-EGFP and FtsZ_C-terminal-mCherry, respectively, and the black line represents the cross-correlation function. **A.** In the absence of GTP and the presence of DMSO as a vehicle, cross-correlation was not observed. **B.** In the presence of both GTP and DMSO, cross-correlation was observed. **C.** In the presence of both GTP and chemical #610, the cross-correlation amplitude was reduced from that in **B**.
(TIF)

S2 Fig Results of 2nd FCCS screening. **A.** Enlargement of the area around chemical #610 shown in the graph of Fig 4. The red line indicates the average binding ratio obtained after addition of DMSO and GTP (= 0.411). The chemicals #607, #608, #609, and #610 had significant inhibitory effects on dimerization of FtsZ. Statistical analysis was performed using student t-test (**P<0.001; *P<0.05). **B.** The structures of chemicals #606–611.
(TIF)

S3 Fig Enlarged graph showing the relationship between the inhibition % and dissociation constant (Kd). (TIF)
