# Supplementary figures and images for: Screening for FtsZ Dimerization Inhibitors Using Fluorescence Cross-Correlation Spectroscopy and Surface Resonance Plasmon Analysis
Source: PLoS One. 2015 Jul 8;10(7):e0130933. doi: 10.1371/journal.pone.0130933 (PMC4496089; doi:10.1371/journal.pone.0130933)

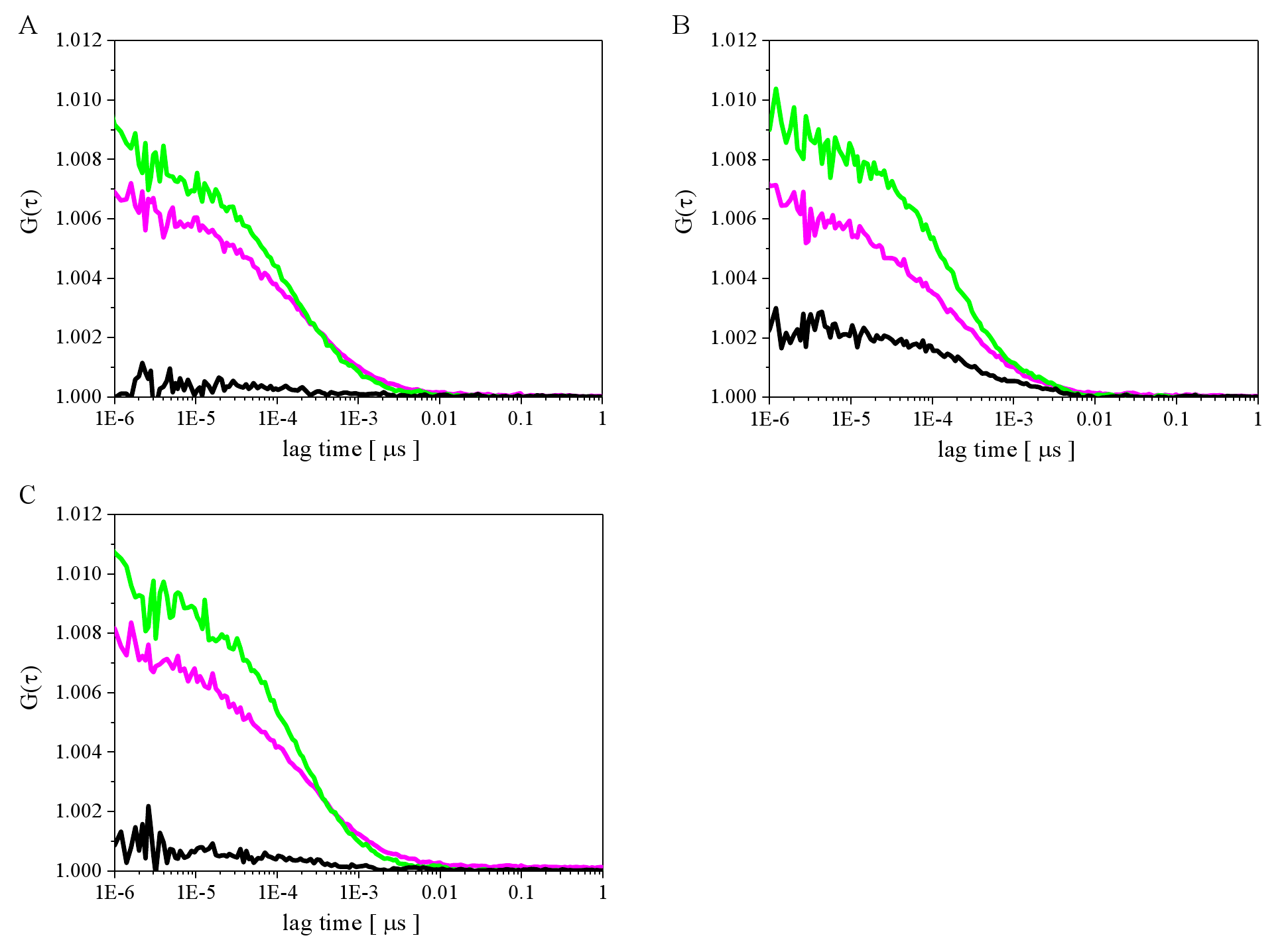

Supplement: S1 Fig — The green and magenta lines represent the autocorrelation functions of FtsZK175D_N-terminal-EGFP and FtsZ_C-terminal-mCherry, respectively, and the black line represents the cross-correlation function. A. In the absence of GTP and the presence of DMSO as a vehicle, cross-correlation was not observed. B. In the presence of both GTP and DMSO, cross-correlation was observed. C. In the presence of both GTP and chemical #610, the cross-correlation amplitude was reduced from that in B. (TIF) [file pone.0130933.s001.tif]

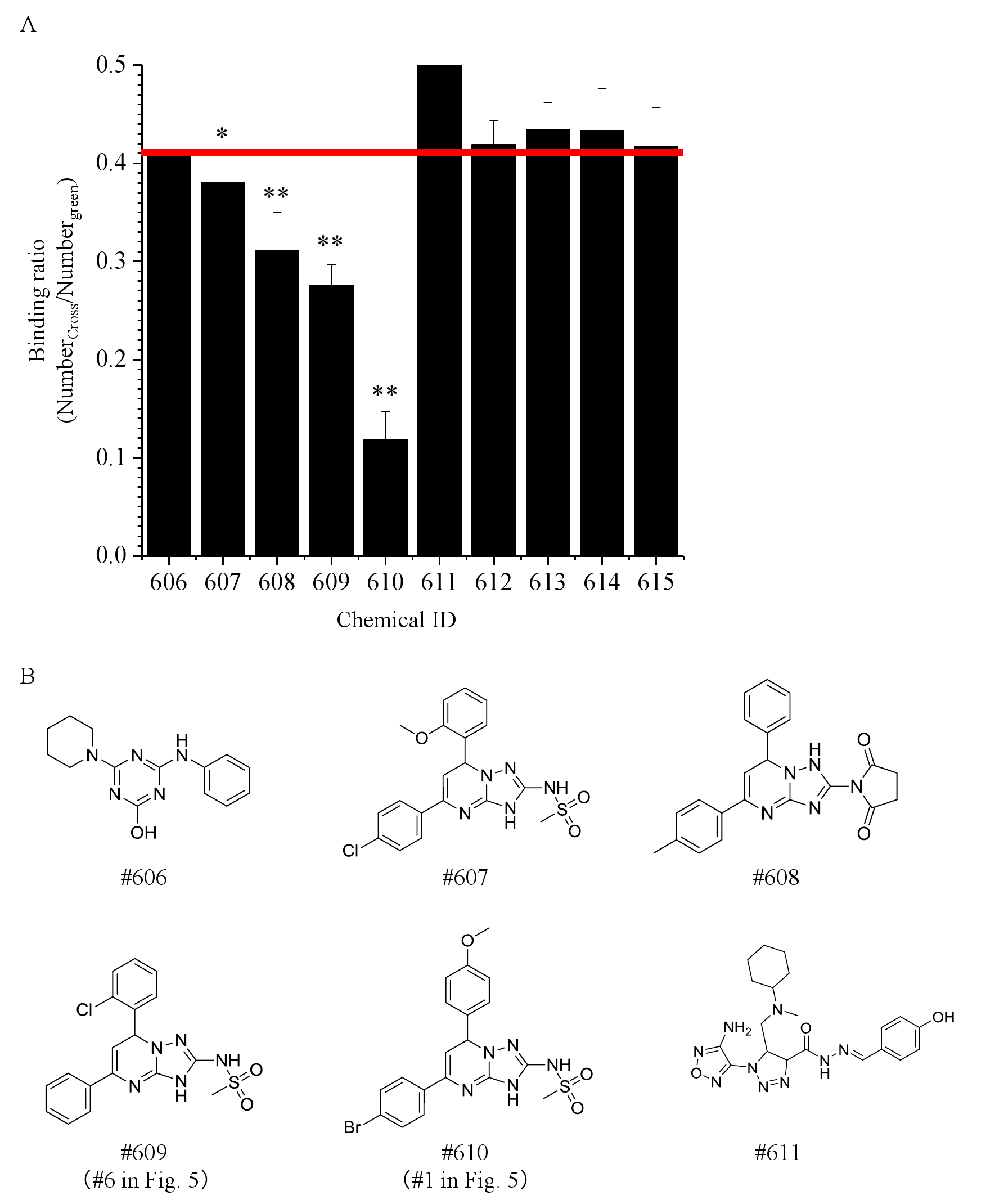

Supplement: S2 Fig — A. Enlargement of the area around chemical #610 shown in the graph of Fig 4. The red line indicates the average binding ratio obtained after addition of DMSO and GTP (= 0.411). The chemicals #607, #608, #609, and #610 had significant inhibitory effects on dimerization of FtsZ. Statistical analysis was performed using student t-test (**P<0.001; *P<0.05). B. The structures of chemicals #606–611. (TIF) [file pone.0130933.s002.tif]

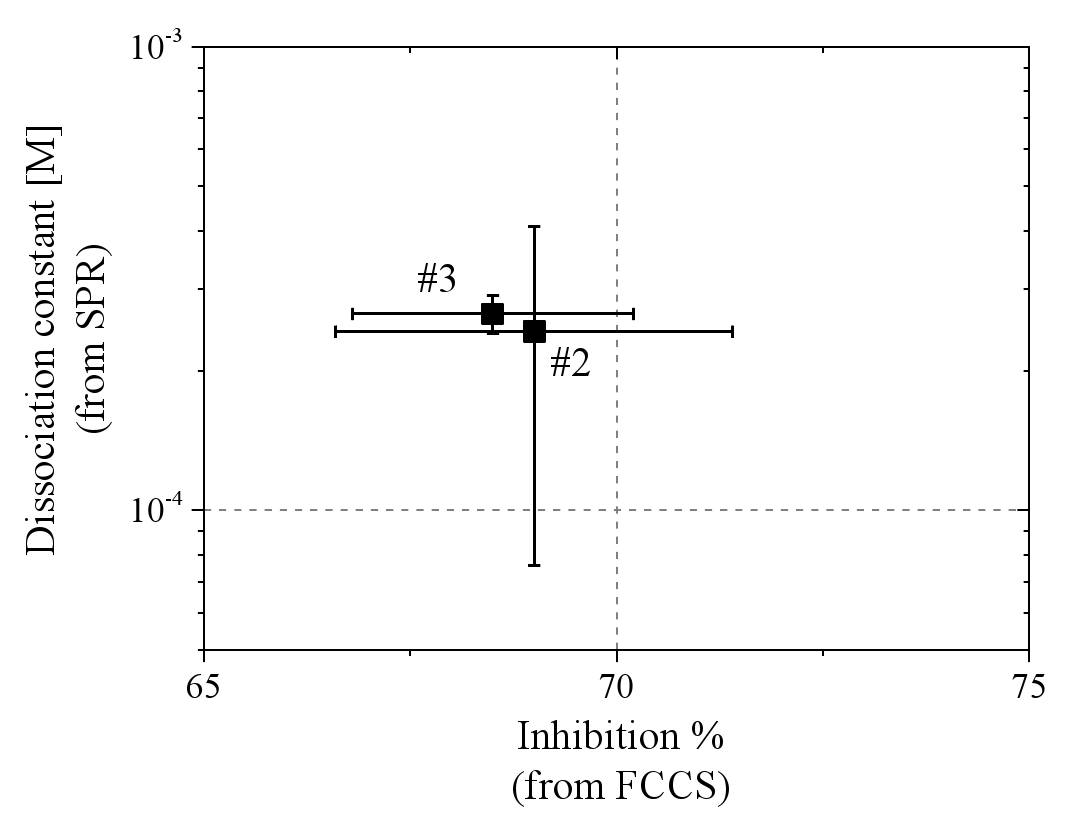

Supplement: S3 Fig — (TIF) [file pone.0130933.s003.tif]
